# The job satisfaction and burnout levels of primary care health workers in the province of Malatya in Turkey

**DOI:** 10.12669/pjms.313.6795

**Published:** 2015

**Authors:** Ozlem Cagan, Osman Gunay

**Affiliations:** 1 Dr. Ozlem Cagan, Eskisehir Osmangazi University, Eskisehir School of Health, Eskisehir 26480, Turkey; 2 Prof. Dr. Osman Gunay, Erciyes University Faculty of Medicine, Department of Public Health, 38039, Kayseri, Turkey

**Keywords:** Primary health care, Health workers, Job satisfaction, Burnout

## Abstract

**Objective:**

The objective was to determine the job satisfaction and burnout levels of primary care health workers in Malatya in Turkey.

**Methods:**

The sample of the study included 186 physicians, 126 midwives and 106 nurses working in primary health care. The Minnesota Job Satisfaction Scale and the Maslach Burnout Scale were used in the study.

**Results:**

The general, internal and external job satisfaction score medians of the study group were 3.35, 3.50 and 3.12 respectively, while the median of the Maslach Personal accomplishment score was 23.00, the Emotional Burnout score median was 15.00, and the Depersonalisation score median was found to be 3.00.

**Conclusions:**

The manner of their employment in the departments where they work, their perception of their economic circumstances and their satisfaction of the department where they work have an impact on the job satisfaction and burnout levels of workers.

## INTRODUCTION

The medical workforce constitutes the foundation of the provision of health services in all countries. The effectiveness of health systems and the quality of health services are directly related to the performance of health workers. Thus, the medical workforce, which is defined as the human face of health systems, possesses a very important place in health services.^1^

The family medicine model was implemented in primary health care services in Turkey in 2004.^2^,^3^ Community Health Centres (CHC) and Family Health Centres (FHC) were set to provide primary health care services. Family physician and family health officers (midwives, nurses and health officers) work together in FHCs. Within the family physician arrangement, where an average of 3.500 people are looked after by one physician and one health officer, the family physician and family health officers carry the responsibility of providing the individuals who are registered with them, with preventative health services and primary care diagnosis, treatment and rehabilitation services. On the other hand, community health centres are organisations which provide preventative, curative and rehabilitating services directed at the community and the environment, under the direction of the public health directorate, monitor, evaluate and assist the efficient provision of these services, and ensure coordination between the health organisations and the other authorities and organisations in their regions. CHCs employ physicians, nurses, midwives, environemotional health technicians and other medical personnel.^1^,^4^

The objective of this study was to determine the job satisfaction and burnout levels of the health workers employed in family health centres and community health centres in the province of Malatya in Turkey.

## METHODS

This study was conducted on the health workers at the family health centres and community health centres in Malatya between August 15 to – October 15, 2011. There were 142 physicians, 89 midwives, 72 nurses employed in the family health centres and community health centres in Malatya city centre, and 71 physicians, 64 midwives and 56 nurses employed in the districts of the province. 186 of a total of 213 physicians, and 232 of a total of 281 midwives and nurses were reached.

### Instruments

The first section consists of 16 questions, comprised of the various socio-demographic characteristics and various characteristics related to their employment status of the health workers.

The Minnesota Job Satisfaction Scale was developed by Weiss et al in 1967, with the purpose of determining the level of job satisfaction.^5^ It was translated into Turkish by Baycan (1985), and studies were carried out into its validity and reliability (Cronbach’s alpha=0.77). The short form of the Minnesota Job Satisfaction Scale, which possesses characteristics revealing the internal and external satisfaction factors of the scale, is a 5-point Likert type scale, consisting of 20 clauses. The scores range between 1.0 and 5.0. Higher scores mean higher job satisfaction.^6^

The Maslach Burnout Scale was developed by Christina Maslach, Susan E. Jackson and Cary Cherniss.^7^ The scale is comprised of 22 questions. It was first translated into Turkish by Ergin^8^ in 1992, and a study was conducted into its validity and reliability. The scale measures three components of burnout; emotional exhaustion (EE), depersonalization (D) and personal accomplishment (PA). High scores on the EE or DP subscales are indicative of burnout, as are low scores on the PS subscale. The study was approved by the Ethics Committee of the Faculty of Medicine of Malatya Inonu University. (Number: 117, Date: 02.08.2011)

The data which was obtained has been assessed on computer, with the help of the SPSS (version 20.0) Statistical Package programme. The conformity of the data to a normal distribution were tested using the Shapiro-Wilk test. The Mann Whitney U test and Kruskal Wallis Variance analysis (Post Hoc Dunn’s test) were used for the statistical analysis of the data which did not display a normal distribution, while the Pearson chi-square test was used for categorical data. The statistical significance was accepted at the level of p<0.05.

## RESULTS

Of those who took part in the study, 66.7% were women, and 33.3% were men. Their ages ranged from 21-60, with the mean age being 36.6±6.3. 8.4% of participants had children. When the professions of the individuals who took part in the study are looked at, it can be seen that 44.4% were physicians and 55.6% were midwives and nurses. 62.2% of those who took part in the study stated that they were satisfied with their jobs.

The gender, age, profession and department worked of those who constituted the work group were not found to have a significant influence on their job satisfaction scores. However, as opposed to this, it was found that the job satisfaction scores of those who perceived their economic status as being poor, who were not happy in their jobs, and who had not chosen their department themselves, were at significantly lower levels (Table-I).

**Table-I:** The distribution of the Minnesota Job Satisfaction Scale Scores of health workers according to their socio-demographic characteristics.

	Minnesota Scale Scores		
	General Job Satisfaction (Median/Min-Max)	Internal Job Satisfaction (Median/min-max)	External Job Satisfaction (Median/min-max)
*Gender*			
Male (n: 139)	3.40 (1.25-5.00)	3.58 (1.17-5.00)	3.13 (1.25-5.00)
Female (n:279)	3.35 (1.00-5.00)	3.42 (1.00-5.00)	3.13 (1.00-5.00)
	Z=0.429, p=0.668	Z=0.981, p=0.327	Z=0.509, p=0.611
*Age Groups*			
≤29 (n:41)	3.35 (1.00-4.30)	3.30 (1.00-5.00)	3.40 (1.00-4.80)
30-39 (n:246)	3.50 (1.00-4.67)	3.41 (1.00-5.00)	3.58 (1.00-5.00)
≥40 (n:131)	3.25 (1.00-4.25)	3.12 (1.00-5.00)	3.12 (1.00-5.00)
	KW =2.205, p=0.332	KW=2.318, p=0.314	KW=1.595, p=0.450
*Profession*			
Physician (n:186)	3.35 (1.25-5.00)	3.58 (1.17-5.00)	3.12 (1.25-5.00)
Midwife (n:126)	3.32 (1.00-5.00)	3.41 (1.00-5.00)	3.12 (1.00-5.00)
Nurse (n:106)	3.35 (1.00-4.85)	3.50 (1.00-4.92)	3.12 (1.00-4.75)
	KW=0.988, p=0.610	KW= 3.445, p=0.179	KW=0.325, p=0.850
*Economic Status*			
Poor (n:34)	2.57 (1.00-4.20)	3.25 (1.00-5.00)	3.45 (1.00-5.00)
Medium (n:194)	2.75 (1.00-4.17)	3.50 (1.00-5.00)	3.62 (1.00-5.00)
Good (n:190)	2.43 (1.00-4.25)	3.12 (1.00-5.00)	3.25 (1.00-5.00)
	KW =23.372, p<0.001	KW=21.195, p<0.001	KW=18.245, p<0.001
*Was primary care work their own personal choice?*			
Yes(n:358)	3.40 (1.00-5.00)	3.58 (1.00-5.00)	3.25 (1.00-5.00)
No(n:60)	2.90 (1.00-4.45)	3.00 (1.00-4.67)	2.68 (1.00-4.25)
	Z=4.851, p<0.001	Z=4.243, p<0.001	Z=4.811, p<0.001
*Satisfaction in the job*			
Satisfied(n:260)	3.60 (1.00-5.00)	3.75 (1.00-5.00)	3.37 (1.00-5.00)
Not satisfied(n:158)	2.90 (1.00-4.80)	3.08 (1.00-5.00)	2.62 (1.00-4.50)
	Z=10.170, P<0.001	Z=9.835, P<0.001	Z=9.158, P<0.001

**Table-II:** The distribution of the Maslach Burnout Scale Scores of health workers according to their socio-demographic characteristics.

Maslach Burnout Scale Scores			
	Emotional Burnout (Median/Min-Max)	Personal Accomplishment (Median/Min-Max)	Depersonalisation (Median/Min-Max)
*Gender*			
Male (n:139)	14.00(0.00-36.00)	15.00(0.00-36.00)	4.00(0.00-19.00)
Female (n:279)	24.00(0.00-32.00)	23.00(0.00-32.00)	3.00(0.00-20.00)
	Z=1.108, p=0.268	Z=0.092, p=0.927	Z=1.488, p=0.137
*Age Groups*			
≤29 (n:41)	14.00 (0.00-32.00)	15.00 (0.00-36.00)	15.00 (0.00-36.00)
30-39 (n:246)	22.00^a^ (5.00-31.00)	23.00^a^ (0.00-32.00)	25.00^b^ (0.00-32.00)
≥40 (n:131)	4.00 (0.00-19.00)	3.00 (0.00-18.00)	3.00 (0.00-20.00)
	KW=0.388, p=0.824	KW=8.226, p=0.016	KW=2.945, p=0.229
*Marital Status*			
Single (n:45)	13.00 (0.00-31.00)	24.00 (8.00-31.00)	4.00 (0.00-19.00)
Married (n:364)	15.00 (0.00-36.00)	23.00 (0.00-32.00)	3.00 (0.00-20.00)
Other (n:9)	10.00 (3.00-24.00)	21.00 (10.00-29.00)	3.00 (0.00-5.00)
	KW=2.503, p=0.286	KW=1.578, p=0.454	KW=3.095, p=0.213
*Profession*			
Physician (n:186)	15.00 (0.00-36.00)	14.00 (0.00-36.00)	15.00 (0.00-35.00)
Midwife (n:126)	23.50 (0.00-32.00)	24.00 (0.00-32.00)	23.00 (5.00-32.00)
Nurse (n:106)	3.00 (0.00-17.00)	3.00 (0.00-20.00)	4.00 (0-19.00)
	KW=0.527, p=0.768	KW=1.242, p=0.537	KW=2.062, p=0.357
*Economic Status*			
Poor (n:34)	22.50 (4.00-36.00)	24.00 (6.00-32.00)	4.50 (0.00-17.00)
Medium (n:194)	15.00 (0.00-36.00)	23.00 (0.00-32.00)	3.00 (0.00-19.00)
Good (n:190)	14.00 (0.00-36.00)	23.00 (0.00-32.000)	3.00 (0.00-19.00)
	KW=15.055, p=<0.001	KW=0.463, p=0.793	KW=6.535, p=0.038
*Was primary health care work their own personal choice?*			
Yes (n:358)	14.00 (0.00-36.00)	23.00 (0.00-32.00)	3.00 (0.00-18.00)
No (n:60)	21.00 (0.00-36.00)	23.00 (12.00-32.00)	3.50 (0.00-20.00)
	Z=3.454, p<0.001	Z=0.725, p=0.468	Z=1.203, p=0.229
*Satisfaction in the job*			
Satisfied (n:260)	11.00 (0.00-33.00)	24.00 (0.00-32.00)	2.00 (0.00-16.00)
Not satisfied (n:158)	22.00 (2.00-36.00)	23.00 (6.00-32.00)	6.00 (0.00-20.00)
	Z=10.470, p<0.001	Z=1.602, p=0.109	Z=6.729, p<0.001

Gender, marital status, age and profession were not found to have a significant influence on their burnout scores. While the personal accomplishment scores of those who worked in districts were significantly higher, it was also found that the emotional burnout scores of those who perceived their economic status to be poor, or those who had not personally chosen the department where they worked, were significantly higher. The emotional burnout and depersonalisation scores of those not happy in their jobs were also found to be high.

## DISCUSSION

The general, internal and external job satisfaction median scores of the primary care health workers employed in Malatya were found to be 3.35 (min;1.00-max;5.00), 3.50 (min;1.00-max;5.00) and 3.12 (min;1.00-max;5.00), respectively. Hagopian has found similar results in Uganda^9^, and Leshabariin Tanzania.^10^

No significant difference has been found in terms of the median job satisfaction scores between men and women, in our study. In the study they conducted on physicians, Voltmer et al.^11^, Dossary et al.^12^, Rosta et al.^13^ and Siu et al.^14^ and in the study conducted on primary care workers, Yavuzyilmaz et al.^15^ have also stated that there is no relationship between jab satisfaction and gender, similar to our findings.

No significant difference has been found in terms of the job satisfaction scores among age groups, in our study. Similar results have been reported by Voltmer et al.^10^ and Sunter et al.^16^ in their studies on practitioner physicians, and by Kurcer et al.^17^ in their study conducted at a faculty of medicine. No significant difference has been found in terms of the job satisfaction scores in connection with gender, age, marital status and professions, in our study.

The job satisfaction scores of those who assessed their economic circumstances as being poor were found to be significantly lower. These results show similarities with the study conducted in Tanzania.^10^ Another organisational characteristic, which has an impact on job satisfaction – salary –is fundamentally directed at meeting the physiological and security needs of workers. It can be considered that an individual who is happy outside of work – as a result of an increase in their quality of life, caused by an increase in their satisfaction related to their income levels – is also more likely to reach satisfaction at work.

The general, internal and external job satisfaction scores among those who have themselves chosen to work at primary care, are significantly high. As working in departments and areas they themselves desire will increase the morale and motivation of individuals, this can also be assessed as being a situation which may be expected to reflect positively on their job satisfaction levels.

The job satisfaction scores of individuals who are happy in their work have been found to be significantly high. In the study carried out by Shi et al.^18^, the job satisfaction levels of 52.4% of primary health care workers was found to be significantly low, while the job satisfaction levels of those who stated that they were happy in their work was found to be higher. It is expected that as long as the nature of the work meets the needs and expectations of those who are carrying out the work, they will derive job satisfaction.

In our study, no relationship was found between professions and burnout scores. Similarly, no difference was found also in the study carried out by Ogresta^19^, on health workers. However, it has been determined in the studies by Pejuskovic et al.^20^, Arigoni et al.^21^ and Goehring et al.^22^ on physicians, that when the emotional burnout scores are high, and by Sharma et al.^23^ and Rachiotis et al.^24^ in their studies, that the emotional burnout levels of nurses is higher than that of doctors. No difference was found between gender and marital status and burnout scores, in our study.

The personal accomplishment scores of workers who are aged 40 and above were found to be significantly high. In their studies, Sharma et al.^23^ and Ahola et al.^25^ have reported that the depersonalisation score rises with age. However, on the contrary, in their studies, Kurcer et al.^17^ and Guduk et al.^26^ have found no difference between age groups, in terms of burnout.

The emotional burnout and depersonalisation scores of individuals, whose economic circumstances are poorer, are higher. As is claimed in many studies, the belief that burnout will fall and job satisfaction will rise together with the income levels of workers increasing, is at a parallel with the results of the studies which have been looked at. The emotional burnout scores of those who did not choose to work at FHCs/CHCs themselves are significantly high.

## CONCLUSION

According to the data obtained from this study, it has been found that the job satisfaction levels of those whose economic circumstances are poor, who are not happy in their work, and who did not choose to work at primary health care, themselves, are low. On the other hand, the burnout scores of those whose economic circumstances are poor, who are not happy in their work, who did not choose to work at primary health care, and who work in city centres have been found to be higher. It has been shown that as the job satisfaction levels of health workers rise, their burnout levels fall.
